# Nursing Interventions in Approaching Trauma Victims: Scoping Review

**DOI:** 10.3390/jcm14093016

**Published:** 2025-04-27

**Authors:** Sofia Padinha, Júlio Belo Fernandes, Cidália Castro

**Affiliations:** 1 Department of Nursing, Almada-Seixal Local Health Unit, 2805-267 Almada, Portugal; 2 Nurs* Lab, Monte da Caparica, 2829-511 Almada, Portugal; jfernandes@egasmoniz.edu.pt; 3 Egas Moniz Center of Interdisciplinary Research (CiiEM), Egas Moniz School of Health & Science, Monte da Caparica, 2829-511 Almada, Portugal

**Keywords:** nursing, intervention, strategy, techniques, methods, polytrauma, multiple trauma

## Abstract

**Background:** Trauma is a leading cause of morbidity and mortality worldwide, often resulting in devastating physical, psychological, and social consequences. Nurses play an essential role in stabilizing patients, managing acute care, and ensuring continuity of treatment. Given the complexity of trauma care, continuous specialized training in nursing is crucial to enhance the quality of interventions and improve patient outcomes. **Objective**: We aimed to map and analyze nursing interventions in approaching trauma victims. **Methods**: This scoping review followed the methodology proposed by the Joanna Briggs Institute. The literature search was conducted in databases available on the EBSCOhost platform and in PubMed. The research question guiding this review was as follows: what nursing interventions are used to approach trauma victims? **Results**: Thus, 1454 articles were identified (348 from ESBOhost and 1106 from PubMed), with 13 meeting the inclusion criteria. The findings were categorized into six key areas: (1) Triage, (2) Initial Approach, (3) Secondary Approach, (4) Professional Training, (5) Interdisciplinary Collaboration, and (6) Care Maintenance. **Conclusions:** Trauma victims require immediate and complex care. Nurses are pivotal throughout all clinical phases, delivering physical and psychological support, collaborating with multidisciplinary teams, and advancing professional training and community education.

## 1. Introduction

Trauma originates from a Greek word meaning “wound”. In medicine, it refers explicitly to injuries caused by external factors [1]. Severe traumatic events involving a high transfer of energy, such as traffic accidents, falls, and gunshot wounds, often result in multiple serious injuries, leading to a polytraumatized person [2].

Beyond its impact on individuals, trauma presents significant social and economic challenges. Therefore, societies must prioritize prevention, treatment, and long-term care, focusing on health. In this context, trauma poses a significant challenge for emergency medicine, critical care, and catastrophe medicine, necessitating specialized surgical and medical responses [3].

According to the World Health Organization [4], approximately 16,000 people die from trauma daily, and thousands more suffer injuries, many with lasting consequences. In Europe, 20,640 people died in road accidents in 2022, with pedestrians, cyclists, and motorcyclists at exceptionally high risk [5]. Trauma remains the leading cause of death worldwide among individuals aged 4 to 44 [6].

Moreover, the long-term social impact of trauma is considerable, as individuals often experience permanent physical, psychological, and social impairments, leading to reduced quality of life and an increased reliance on healthcare and social support systems. Studies have shown that individuals who experience trauma-related social dysfunction are more likely to screen positive for post-traumatic stress disorder, to have persistent functional limitations, and to face difficulties in returning to work [7].

Additionally, a study by van der Vlegel et al. [8] investigated the impact of the injury severity level on healthcare utilization and costs, identifying key predictors for both healthcare and productivity expenses. On average, patients with paid employment returned to work 21 weeks after injury. The mean total cost per patient was EUR 24,760, broken down into EUR 11,930 for in-hospital care, EUR 7770 for post-hospital services, and EUR 8800 in productivity losses. An Injury Severity Score (ISS) ≥ 25 and lower health status were associated with significantly higher healthcare costs, while male sex was linked to increased productivity costs. These findings reinforce the substantial economic burden of trauma and the importance of effective prevention, early intervention, and coordinated post-acute care.

Considering the high incidence of trauma-related events, healthcare professionals must adopt a structured and systematic approach to assessing trauma victims to ensure optimal care [9]. A crucial concept in trauma management is the golden hour, which emphasizes timely resuscitation and early injury identification. This concept emerges as the basis of the methodology presented in Advanced Trauma Life Support (ATLS), initially developed by the American College of Surgeons and the Committee on Trauma, which is now widely implemented in trauma centers [10].

The development of rapid trauma intervention systems can significantly reduce early mortality by enabling prompt, appropriate treatment. Additionally, a deeper understanding of multiorgan failure and post-trauma complications, such as infections, can help prevent late deaths [11].

Nurses play a fundamental and multifaceted role within this complex and dynamic context [2]. As frontline responders in emergency departments and trauma units, nurses are often the first healthcare professionals to assess and stabilize individuals affected by trauma [12]. Their responsibilities include rapid triage, monitoring vital signs, administering medications, managing wounds, providing emotional support, and coordinating care across multidisciplinary teams [4,12].

Beyond technical competencies, trauma nursing requires the capacity to deliver holistic, person-centered care that addresses both the physiological and psychological dimensions of trauma. Nurses provide continuity of care from the acute phase through rehabilitation to recovery, acting as patient advocates and educators for individuals and their families [13,14].

Given the increasing complexity of trauma care and nurses’ evolving roles, there is a need to better understand the specific interventions they employ in practice. Mapping these interventions can inform clinical guidelines, support training and education, and promote high standards of trauma care delivery.

Therefore, this scoping review aims to systematically identify, map, and analyze the range and characteristics of nursing interventions used in the care of trauma victims.

## 2. Methodology

The current scoping review was conducted according to the Joanna Briggs Institute [15] methodology. To enhance transparency and maintain the overall quality of the review, the Preferred Reporting Items for Systematic Reviews and Meta-Analyses Extension for Scoping Reviews (PRISMA-ScR) checklist was applied to the report [16]. The protocol for this scoping review was not registered.

The research question was formulated using the mnemonic ’PCC’, where P represents the participants, C represents the concept, and C represents the context under study. The research question is as follows: what nursing interventions are used in the approach to trauma victims?

### 2.1. Criteria for Inclusion

The inclusion and exclusion criteria were established following the PCC mnemonic (Table 1).

All studies not meeting the defined inclusion criteria were eliminated from this scoping review.

### 2.2. Search Strategy

The following Medical Subject Headings (MeSH) were used to construct the search string: Nurs* AND (interventions OR strategies OR techniques OR methods) AND (polytrauma OR multiple trauma). A search was conducted in the PubMed database and the EBSCOhost platform, including MEDLINE Complete, CINAHL Complete, Nursing & Allied Health Collection: Comprehensive, Cochrane Central Register of Controlled Trials, and MedicLatina.

### 2.3. Study Selection and Data Extraction

**Figure 1:**
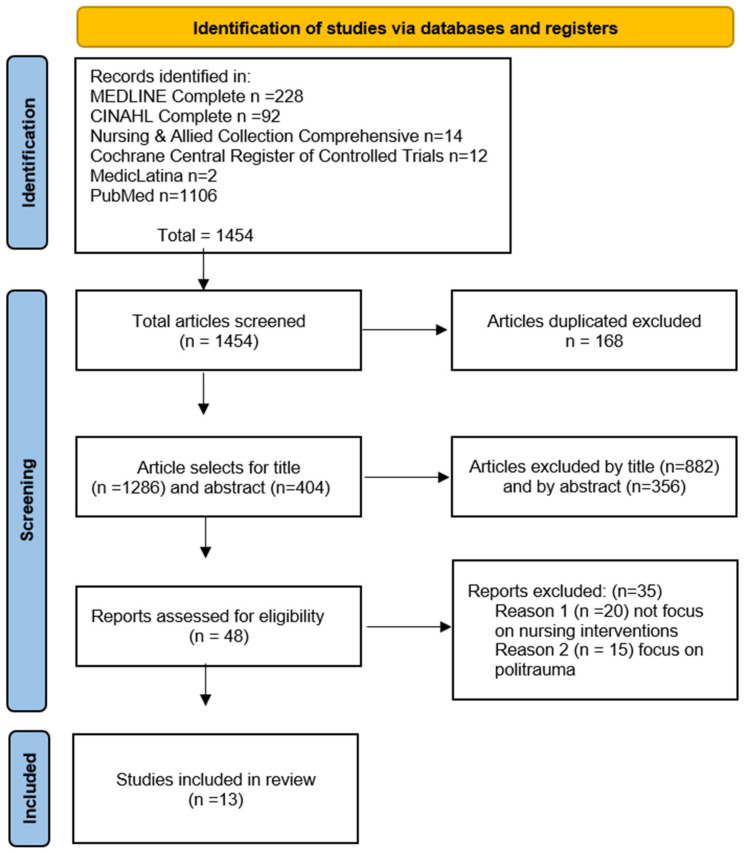
PRISMA flowchart for study selection.

The study selection process was conducted using Rayyan, an AI-powered systematic review management platform. Two independent reviewers (S.P. and C.C.) performed the screening process in three sequential phases: initial title screening, followed by abstract review, concluding with full-text assessment. When uncertainty about a study’s relevance arose at each stage, reviewers adopted an inclusive approach by advancing the article to the next phase. To ensure methodological rigor, disagreements between the primary reviewers were resolved through consultation with a third reviewer (J.B.F.), who made the final determination. This comprehensive screening procedure, completed on 26 January 2024, identified studies meeting our predefined inclusion criteria. The complete selection process, including the number of articles excluded at each stage, is visually represented in the PRISMA flow diagram (Figure 1), which documents the progression from identification through screening and eligibility assessment to final inclusion.

The research team systematically extracted and organized all relevant data into a comprehensive table (Table 2) for thorough analysis. This structured approach followed a consistent format that included each study’s author(s), the publication year, the complete article title, the country where the research was conducted, the study’s aims, the methodological approach employed, and the nursing interventions described or evaluated.

## 3. Results

The search strategy yielded an initial pool of 1454 articles sourced from two primary databases: 1106 articles from PubMed and 348 from EBSCOhost. The EBSCOhost results comprised publications from several specialized collections: 228 from MEDLINE Complete, 92 from CINAHL Complete, 14 from Nursing & Allied Health Collection: Comprehensive, 12 from the Cochrane Central Register of Controlled Trials, and 2 from MedicLatina. The selection process ultimately identified 13 articles published between 2006 and 2024 that met our inclusion criteria, all focusing on nursing interventions for trauma victims in critical situations.

The selected studies represented diverse geographical origins, with five originating from the United States [17,18,19,20,21], three from Brazil [22,23,24], two from England [25,26], and one each from China [27], Switzerland [28], and South Korea [29]. Methodologically, the corpus included four integrative reviews [17,23,24,25], three descriptive studies [18,21,26], two literature reviews [20,27], and one each of the following: a case study [19], a qualitative comparative descriptive study [22], a prospective study [28], and a descriptive-correlational study [29].

Table 2 summarizes the included articles, consolidating their most significant characteristics and results.

**Table 2:** Data extraction and synthesis.

Author/Year/Title/Country	Aim	Methodology	Interventions/Categories
Laskowski—Jones [17], 2006 Responding to trauma: your priorities in the first hour USA	Identify and summarize interventions for trauma victims.	Integrative Review	**Triage:** -Preparation of the emergency service and their professionals; -Previous knowledge of the situation; -Professionals ready to act and with appropriate Personal Protective Equipment; -Means of diagnostic and therapeutic in pre-warning. **Initial Approach:** -CABCDE (C: Catastrophic Haemorrhage, A: Airway, B: Breathing, C: Circulation, D: Neurological Dysfunction, E: Exposure). **Secondary Approach:** -More detailed assessment (cephalic—caudal); -AMPLE (A: Allergies, M: Medication, P: Past History, L: Last meal, E: Events); -Provide definitive care; -Transfer of the person to a permanent unit.
Anderson & Watson [18], 2013 Traumatic Shock: The Fifth Shock USA	Demonstrate the importance of approaching trauma like the 5th shock.	Descriptive study	**Initial Approach:** -Supportive therapy. **Secondary Approach:** -More detailed assessment; -Interventions associated with possible injuries.
Crossan & Cole [25], 2013 Nursing challenges with a severely injured patient in critical care England	Understand the complex issues in treating a person in a critical situation, victim of trauma	Integrative Review	**Initial Approach:** -CABCDE (C: Catastrophic Haemorrhage, A: Airway, B: Breathing, C: Circulation, D: Neurological dysfunction, E: Exposure) **Secondary Approach:** -Knowledge of the lethal Triad.
Stewart [19], 2014 Blunt Chest Trauma USA	Understand the pathophysiology, diagnosis and treatment of thoracic trauma and nursing interventions in trauma.	Case study	**Initial Approach:** -ABCDE (Targeted interventions such as the ABC in thoracic trauma).
Stanford et al. [26], 2016 Assessment of injury severity in patients with major trauma England	Synthesize information about care provided in trauma centers, and which protocols are most used.	Descriptive study	**Triage:** -Manchester triage; -Prioritize care interventions; -Identify injury mechanisms and degree of instability. **Initial Approach:** -CABCDE (C: Catastrophic Haemorrhage, A: Airway, B: Breathing, C: Circulation, D: Neurological dysfunction, E: Exposure); -Glasgow coma scale; -Assessment of pain. **Professional training:** -Injury Severity Score (ISS) provides nurses with the degree of injury suffered by the trauma; -Continuous training to acquire skills. **Interdisciplinary Collaboration:** -Provision of physical and mental care among the multidisciplinary team; -Care is centered on the person who is the victim of trauma.
Trecossi et al. [22], 2018 Educational interventions on initial hospital care to polytraumatized Brazil	Compare two nursing intervention methodologies on initial hospital care for polytraumatized patients	Quantitative, comparative and descriptive study	**Professional training:** -Knowledge of trauma interventions based on the Advance Trauma Care for Nurses (ATCN); -Knowledge of internal hospital protocols. **Interdisciplinary Collaboration:** -Communication between different professional groups; -Teamwork.
Liu et al. [20], 2019 Trauma response nurse: Bringing critical care experience and continuity to early trauma care USA	Understand the benefits of specialized trauma intervention by nurses and the benefits achieved.	Literature Review	**Initial Approach:** -Assist in clinical interventions. **Secondary Approach:** -Transport trauma victims from the emergency department to diagnostic exams, procedures or different units. **Professional training:** -Peer education; -Promote trauma programs; -Ensure that trauma victims receive the best possible trauma care; -Ensure contact with the community by promoting health education. **Interdisciplinary Collaboration:** -Promote improvement in the care provided by every professional group.
Zang et al. [27], 2019 Combined penetrating trauma of the head, neck, chest, abdomen, and scrotum caused by falling from a high altitude: A case report and Literature Review China	Understand and synthesize possible interventions for victims of trauma due to a fall from altitude.	Literature Review	**Triage:** -Knowledge of injury mechanisms; -Evaluation of vital signs and general condition of the person; -Referral and forwarding to the resuscitation room. **Initial Approach:** -CABCDE (C: Catastrophic Haemorrhage, A: Airway, B: Breathing, C: Circulation, D: Neurological dysfunction, E: Exposure); **Secondary Approach:** -More detailed objective examination; -Clinical history. **Interdisciplinary Collaboration:** -Communication between different professional groups; -Provide diagnosis exams; -Multidisciplinary support for the family. **Care Maintenance:** -Prevent complications and infections; -Psychological care for people who are victims of trauma.
Bellino et al. [21], 2020 Owning the Trauma Bay: Teaching trauma resuscitation to emergency medicine residents and nurse’s trough in-situ simulation USA	Evaluate the knowledge and interventions provided by the medical and trauma nursing teams during simulations.	Descriptive study	**Initial Approach:** -CABCDE (C: Catastrophic Haemorrhage, A: Airway, B: Breathing, C: Circulation, D: Neurological dysfunction, E: Exposure); -Assess vital signs and hemodynamic monitoring; -Ensure airway venous access and monitor mental status; -Apply predefined protocols. **Professional training:** -Trauma simulation leads to more knowledge and more confidence in providing care. **Interdisciplinary Collaboration:** -Communication between different professional groups; -Promote teamwork.
Ferreira et al. [23], 2022 Nursing outcomes for patients with multiple trauma and impaired physical mobility: An integrative review Brazil	Identify nursing interventions and activities for victims of multiple traumas with reduced physical mobility	Integrative Review	**Care Maintenance:** -Skin care and maintenance; -Positioning in bed; -Prevention of pressure points; -Assistance in self-care and hygiene; -Assistance in oral hygiene self-care; -Ensure moments to prevent circulatory problems; -Assistance with restricted mobility.
Lattoni et al. [28], 2022 Effect of structured briefing prior to patient arrival on interprofessional communication and collaboration in the trauma team Switzerland	Evaluate interprofessional collaboration and the efficiency of interventions within the trauma team; evaluate the effects of briefing on teamwork before the trauma victim’s arrival	Prospective Study	**Triage:** -Knowledge of injury mechanisms; -Application of briefing before the arrival of the trauma victim. **Interdisciplinary Collaboration:** -Effective communication; -Cooperation in providing care; -Determination of roles (eg: team leader).
Ferreira et al. [24], 2023 Nursing interventions and activities for patients with multiple traumas: An integrative review Brazil	Identify nursing interventions and activities for people victims of multiple traumas with reduced physical mobility	Integrative Review	**Care Maintenance:** -Organize and assist in alternating body positions; -Promote energy conservation; -Monitor intestinal and bladder elimination; -Restrict movements to essential ones to reduce the risk of sequelae; -Ensure adequate nutritional care; -Promote self-care whenever possible.
Kim & Roh [29], 2024 Perceived trauma nursing core competency, interprofessional collaborative competency and associated barriers among regional trauma center nurses South Korea	Determine the importance of trauma nurses’ skills and performance levels and the barriers identified.	Descriptive—Correlational Study	**Professional training:** -Advanced nursing interventions; -Education of peers to promote targeted and practical trauma nursing actions. **Interdisciplinary Collaboration:** -Quick thinking and integrated with other professional groups; -Care provided with interprofessional cooperation.

**Figure 2:**
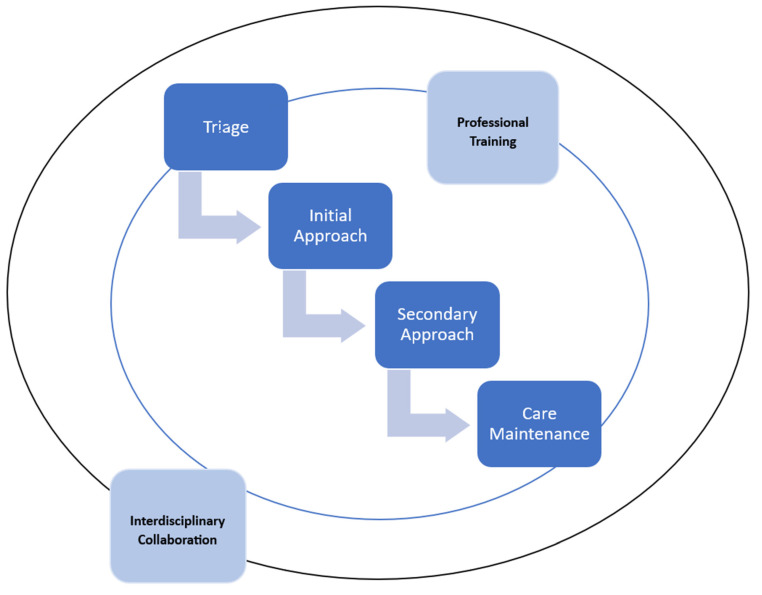
Nursing interventions in trauma care.

The data analysis employed an inductive categorization process to organize and compare the identified nursing interventions systematically [30]. Through this analytical approach, the collected information was grouped into six distinct categories: (1) Triage, (2) Initial Approach, (3) Secondary Approach, (4) Professional Training, (5) Interdisciplinary Collaboration, and (6) Care Maintenance (Figure 2).

### 3.1. Triage

Triage plays a critical role in the initial management of trauma victims, as it determines care priorities and pathways [17]. The arrival of a trauma patient always involves significant uncertainty, requiring both team preparedness and diagnostic resources. Information from pre-hospital teams is particularly valuable for predicting potential injuries and guiding care planning [17].

Adequate team preparation at this stage should include briefings to enhance collaboration and communication among all staff members [28]. Given the high risk of exposure to bodily fluids, especially blood, during trauma assessment, the team must also be equipped with appropriate personal protective equipment (PPE) [17].

In the initial contact, the trauma victim’s assessment is carried out, including objective and subjective observations (such as vital signs assessment) and a neurological evaluation (assessing consciousness level, motor deficits, and pupillary characteristics) [27].

It is essential to gather information regarding the accident’s mechanics and any factors that may explain potential injuries during the information gathering. This information can be obtained through family members or pre-hospital emergency teams [17,26,28].

The Manchester Triage system is commonly used for screening and is an effective tool for prioritizing cases. While it cannot predict the clinical progression of a trauma victim, Triage helps identify individuals most likely to have sustained significant injuries due to the trauma mechanisms [26].

### 3.2. Initial Approach

As soon as a trauma victim is identified, rapid response protocols are activated, and the person is promptly referred to the resuscitation room for specialized treatment [27].

The initial approach follows the CABCDE protocol, which is specific to trauma victims. The first step, catastrophic hemorrhage (C), prioritizes the identification and control of exsanguinating bleeding, significantly reducing mortality. This is followed by the assessment of airway (A), breathing (B), circulation (C), neurological dysfunction (D), and exposure (E). This process implements immediate intervention to address any identified issues [26].

During airway assessment (A), the primary objective is to ensure its patency. This involves checking for foreign bodies (e.g., blood, food, or other external substances) that may obstruct the airway [17,21]. At this stage, if there is a change in the state of consciousness, it may be necessary to use an airway adjuvant as protection to prevent airway obstruction due to tongue displacement, such as an oropharyngeal or nasopharyngeal tube (this last one is only used if there is no facial trauma). If spinal cord trauma is suspected, the cervical spine must be stabilized in a neutral position or with the aid of a cervical collar [17,27]. In severe cases, advanced interventions, such as tracheal intubation or a supraglottic device (e.g., laryngeal mask airway), may be necessary [17,25].

Breathing assessment (B) includes evaluating respiratory pattern, rate, movement amplitude, symmetry, and peripheral oxygen saturation. At this stage, auscultation, percussion, and the presence of pain complaints should be carried out, as well as crepitus or the presence of pulmonary emphysema in the chest and neck, as a way of excluding pneumothorax [17,27]. If a possible pneumothorax or hemothorax is diagnosed, it may be necessary to place a chest tube to drain this content and prevent complications [19]. The nursing team is critical in assisting and supporting these procedures [20].

Circulation assessment (C) requires hemodynamic monitoring, including frequent evaluation of heart rhythm, heart rate, and blood pressure (every 5–15 min or as needed) [17,21,27]. External bleeding must be promptly controlled, while internal bleeding is suspected based on accident kinematics and physical signs. Nurses must establish two large-caliber peripheral venous accesses or intraosseous access if this is not feasible [17,21,25,27]. If there is a need to administer fluid therapy, it must be warmed to prevent hypothermia [17]. Additionally, supportive treatment must be administered [18]. Blood samples should be collected for laboratory analysis (including complete blood count, coagulation profile, and cardiac markers) and blood typing [17,19]. A 12-lead electrocardiogram should be performed to assess ischemic changes or signs of cardiac contusion [19]. Pain management is also crucial at this stage, with the nursing team administering prescribed analgesics to alleviate the significant pain experienced by trauma victims [26].

Neurological dysfunction assessment (D) involves evaluating the level of consciousness using the Glasgow Coma Scale [17,21,26]. Additionally, ocular response (pupillary reflexes, size, shape, symmetry, and reaction to light) and motor response (identification of motor or sensory deficits) should be assessed [17].

Finally, exposure assessment (E) requires a full-body examination. All clothing must be removed—especially if wet—to check for foreign objects and injuries. Body temperature should be monitored using thermal blankets or room heating to prevent hypothermia [17].

### 3.3. Secondary Approach

In the secondary approach, a more detailed assessment is implemented. All injuries are assessed, not just those that are life-threatening. A systematic cephalo-caudal examination is recommended, assessing bruises, lacerations, deformities, and oedema during the objective examination while always considering the person’s pain complaints [17,18,27].

An essential concept in trauma care is the lethal triad, which describes the decompensation caused by significant hemorrhage, leading to three critical factors: hypothermia, coagulopathy, and acidosis. This triad significantly increases mortality and amplifies the inflammatory response. Recognizing and addressing these factors enhance the person’s chances of recovery [25].

All necessary interventions must be carried out to overcome possible complications or sequelae [18]. After the objective examination, it is also essential to collect all clinical information using the AMPLE mnemonic, to allow for recognition of allergies (A), usual medication (M), past clinical history (P), last meal (L) and trauma-related events (E) [17,27].

To conclude this phase, complementary diagnostic tests are performed, definitive treatment is initiated, and the person is transferred to an appropriate care unit, where, based on their clinical condition, they will continue to receive the necessary medical and nursing care [17,20].

### 3.4. Professional Training

Considering the high complexity of care required for trauma victims, healthcare professionals must undergo regular training in this field. Carrying trauma simulations enhances knowledge acquisition, builds confidence, and improves practical skills when managing trauma situations [21,26].

The methodology presented in Advanced Trauma Life Support (ATCN) serves as the foundation for many action protocols, leading to best practices in trauma care [22]. These training programs allow professionals to develop advanced nursing interventions while fostering peer education, resulting in more targeted and effective trauma care [29].

One key concept reinforced in these trainings is the Injury Severity Score (ISS), which enables nurses to assess the extent of injuries and respond accordingly [26]. Beyond immediate trauma management, nurses play a vital role in maintaining community engagement, offering health education, and contributing to better long-term outcomes for trauma victims [20].

### 3.5. Interdisciplinary Collaboration

Trauma care requires seamless interdisciplinary collaboration, with effective communication as the cornerstone for coordinated and high-quality interventions [20,21,22,27,28]. Clearly defining roles, including designating a team leader, ensures efficient teamwork and structured decision making in trauma scenarios [28].

Integrated and rapid coordination among professionals is essential for holistic care addressing the victim’s physical and psychological needs [26,29]. Additionally, supporting the families of trauma victims is a shared responsibility among nurses, physicians, and psychologists, ensuring comprehensive care [27].

### 3.6. Care Maintenance

This final category includes nursing interventions aimed at preventing complications. Psychological distress is a significant concern, as trauma victims often experience long-term emotional and psychological consequences. Additionally, the risk of infection must be proactively managed [27].

To ensure optimal recovery, nurses must focus on skin integrity, proper positioning, pressure ulcer prevention, self-care assistance, hygiene maintenance (including oral hygiene), and safe mobilization to reduce circulatory complications [23].

During later stages of recovery, nursing care should include regular repositioning to minimize pressure injuries, energy conservation strategies, monitoring bowel and bladder function (especially given reduced mobility), controlled movement to prevent long-term impairments, nutritional support, and patient comfort [24].

## 4. Discussion

The analysis of the selected documents identified nursing interventions that directly addressed the research question defined at the outset of this scoping review. The collected data demonstrated a high degree of consistency across most analyzed documents.

A significant portion of the selected studies focused on Triage procedures and the Initial and Secondary Approaches to trauma victims [17,18,19,20,21,25,26,27,28]. Others highlighted nursing interventions related to Professional Training, Interdisciplinary Collaboration, and Care Maintenance [22,23,24,29]. These findings underscore the extensive and multidisciplinary skill set required for nurses working in trauma care.

The reviewed studies span publication trends from 2006 to 2024. While all studies addressed direct interventions (such as Triage, Initial Assessment, and Secondary Evaluation), older publications emphasized these more frequently [17,18,25]. In contrast, more recent studies focused on Care Maintenance, Interdisciplinary Collaboration, and continuous Professional Training, highlighting the evolution of nursing education and trauma care practices [22,23,24,29].

Given that the selected documents originate from three different continents, the findings reflect the global relevance of trauma care. As noted by Kim and Roh [29], trauma remains the leading cause of mortality worldwide. Nurses are pivotal in managing critically injured individuals, being the first healthcare professionals to assess the severity of injuries and initiate appropriate treatment protocols. Evidence suggests that nursing interventions enhance the quality and safety of care, foster interdisciplinary communication, and ensure continuity of care [20].

An interesting finding is the variation in specialized nursing roles across different countries. In international contexts, trauma nurses are designated by various titles, including Trauma Nurse Practitioner [19], Trauma Response Nurse [20], and Trauma-Certified Emergency Nurse [21]. Despite the differences in nomenclature, these roles consistently refer to specialist nurses with advanced skills in trauma management.

This review’s findings reinforce nursing’s fundamental role in stabilizing critically injured individuals. The nursing process begins at Triage, followed by the Initial Approach (CABCDE method) and secondary assessment [17,18,19,20,21,25,26,27,28]. These interventions ensure a comprehensive and timely evaluation supported by all necessary diagnostic tools and procedures.

Over time, nursing care has expanded beyond physical stabilization to include psychosocial support. Nurses now play a key role in holistic care, recognizing the importance of addressing both the physical and psychological well-being of trauma victims. Furthermore, community-based interventions have gained prominence, aiming to educate and prepare the population for emergencies, thereby improving local response capabilities [20].

During the post-stabilization phase, several studies highlight the importance of Care Maintenance, particularly for patients with reduced mobility and loss of independence [23]. Therefore, many of the interventions described as recommended for nursing practice focus on restricting movement only to what is essential to prevent sequelae and avoid pressure ulcers through skin care, maintenance, and position changes [23,24]. These authors also highlight the importance of nursing assistance in promoting self-care whenever possible, particularly regarding hygiene and monitoring intestinal and bladder elimination.

At this stage, Zang et al. [27] emphasize the importance of nurses’ interventions in preventing complications, particularly infections, and stress the psychological care that must be provided to patients.

Furthermore, quality care must always be provided without neglecting pain management. This is a central aspect of nursing practice, and its promotion among peers is crucial [20].

Thus, it can be stated that a person who is a victim of polytrauma requires highly complex care from the nursing team to ensure the resolution of their clinical condition [19].

### Limitations

This scoping review provides significant value by identifying nursing interventions for trauma victims. However, it is essential to acknowledge some limitations inherent to the methodological process adopted.

The selection of databases used may have restricted the scope of this review, potentially excluding relevant studies available from other sources. Additionally, the chosen descriptors may not cover all terminology used in the literature, which could have led to the omission of some pertinent studies. The applied time limit criterion may also represent a limitation, as older but still relevant papers may not have been considered.

Another relevant limitation relates to the geographical diversity of the included studies. This review encompasses articles from different countries, where trauma case patterns can vary significantly due to factors, such as emergency infrastructure, healthcare systems, prevalence of specific types of trauma, and adopted clinical protocols. These variations directly influence the training and preparedness of nursing professionals and the therapeutic approaches used in each context.

As a result, the interventions described in the studies may not be universally applicable or generalizable to all clinical settings, which affects the interpretation and extrapolation of the findings.

Therefore, while this review provides a comprehensive overview of the topic, it is essential to consider these limitations when analyzing its results.

## 5. Conclusions

With trauma becoming increasingly prevalent in clinical practice, nurses must continuously seek scientific evidence to inform their daily practice. Recognizing nurses’ diverse skills underscores their essential role in comprehensive healthcare delivery.

This review highlights a divide in focus among authors—some prioritize the immediate, technical approach to trauma care, while others emphasize long-term Care Maintenance, Interdisciplinary Collaboration, and Professional Training.

Thus, fostering a holistic, person-centered approach in nursing is becoming a priority. This scoping review can catalyze future research, ultimately aiming to enhance care quality through evidence-based practice.

Future studies should focus on developing and validating nursing-sensitive indicators specific to trauma care outcomes, particularly those reflecting long-term recovery and quality of life. Evaluating the effectiveness of nursing interventions in trauma contexts is essential to ensure that care strategies are evidence-based and contribute meaningfully to patient-centered recovery.

## Figures and Tables

**Table 1:** Inclusion/exclusion criteria.

Parameter	Inclusion Criteria
Participants	Nurses who provide care to critically ill patients
Concept	Studies reporting nursing interventions for critically ill patients
Context	Trauma victims
Types of study	All studies available and published in free databases and full-text
Language	No linguistic limit
Time limit	All studies published between 2005 and 2024

## Data Availability

The data presented in this study are available on request from the first author.
